# Effects of a Job Crafting Intervention Program on Work Engagement Among Japanese Employees: A Randomized Controlled Trial

**DOI:** 10.3389/fpsyg.2020.00235

**Published:** 2020-02-21

**Authors:** Asuka Sakuraya, Akihito Shimazu, Kotaro Imamura, Norito Kawakami

**Affiliations:** ^1^Department of Public Health, School of Medicine, Tokyo Women’s Medical University, Shinjuku, Japan; ^2^Faculty of Policy Management, Keio University, Fujisawa-shi, Japan; ^3^Department of Mental Health, Graduate School of Medicine, The University of Tokyo, Bunkyo, Japan

**Keywords:** job crafting, work engagement, mental health, well-being, employee, randomized controlled trial

## Abstract

**Objective:**

The current randomized controlled trial investigated the effectiveness of a job crafting intervention program on work engagement as the primary outcome and job crafting as the secondary outcome among Japanese employees.

**Methods:**

Participants who met the inclusion criteria were randomly assigned to an intervention group (*n* = 138) or a control group (*n* = 143). The job crafting intervention program provided only to the intervention group consisted of two 120-minute group sessions with e-mail or letter follow-up. Outcomes were assessed at baseline and at 3-month and 6-month follow-up in both groups.

**Results:**

In the total sample, the job crafting intervention program showed a non-significant effect on work engagement at both 3-month and 6-month follow-up. Also, job crafting did not improve significantly. However, the program showed a significant intervention effect on work engagement (*p* = 0.04) with small effect size (Cohen’s *d* = 0.33 at 3-month follow-up) of workers in a lower job crafting subgroup.

**Conclusion:**

The job crafting intervention program may not be sufficiently effective to improve work engagement and job crafting for the entire sample of participants. However, it may be effective for workers in lower job crafting subcategories.

**Clinical Trial Registration:**

UMIN Clinical Trials Registry (www.umin.ac.jp/ctr/), identifier UMIN000026668.

## Introduction

### The Importance of Work Engagement

Recently, the field of Occupational Health Psychology has given more attention to the positive aspects of mental health and well-being (Schaufeli and Bakker, 2003; Schaufeli, 2004). One of the most well-known positive mental health outcomes is work engagement, defined as an active, positive, work-related state of mind characterized by vigor, dedication, and absorption (Schaufeli et al., 2002; Bakker et al., 2008). This definition of work engagement guided the development of the Utrecht Work engagement Scale (UWES), which has been used in an increasing number of observational studies measuring work engagement (Schaufeli et al., 2002; Schaufeli and Bakker, 2004; Halbesleben, 2010; Christian et al., 2011).

Previous studies have shown work engagement to be associated with a variety on positive outcomes. Longitudinal studies have reported that work engagement is related to decreased risk of onset of a major depressive episode (Imamura et al., 2016); decreased levels of depressive symptoms (Hakanen and Schaufeli, 2012), psychological distress, and physical complaints (Shimazu et al., 2012, 2015); decreased absence from work due to health issues (Rongen et al., 2014) and medically-certified mental health problems (Roelen et al., 2015); and increased work ability (Rongen et al., 2014). A cohort study found work engagement to be associated with heightened life satisfaction (Hakanen and Schaufeli, 2012); and meta-analyses of cross-sectional and longitudinal studies have found work engagement to be associated with low turnover intention and high employee performance, job satisfaction, organizational commitment, and job involvement (Halbesleben, 2010; Christian et al., 2011). Thus, work engagement could be an important factor in enhancing both health and job-related outcomes of workers.

### The Intervention Studies to Improve Work Engagement

To date, about two dozen intervention studies have been conducted to improve work engagement. A major challenge is a small effect size of these interventions. A meta-analysis of the effectiveness of interventions to increase work engagement based on 14 controlled studies reported a significant but small overall effect size (Hedges *g* = 0.29, 95%-CI = 0.12–0.46) (Knight et al., 2017). However, the meta-analysis included non-randomized controlled trials that provided only a low quality of evidence (Knight et al., 2017). In April 2018, we used the key term “work engagement” to conduct a separate search of the literature on randomized controlled trials (RCTs) using UWES to measure overall work engagement. We found 19 RCTs conducted since 2012 (Hengel et al., 2012; Vuori et al., 2012; Strijk et al., 2013; Ângelo and Chambel, 2013; Coffeng et al., 2014; van Berkel et al., 2014; Imamura et al., 2015, 2017; Bernburg et al., 2016; Buijze et al., 2016; Ebert et al., 2016a, b; Heber et al., 2016; Vallières et al., 2016; van Dongen et al., 2016; Klatt et al., 2017; Michishita et al., 2017; Steinberg et al., 2017; Gollwitzer et al., 2018). However, only six studies showed a significant improvement in work engagement in the intervention group compared to the control group (Imamura et al., 2015, 2017; Ebert et al., 2016a; Heber et al., 2016; Steinberg et al., 2017; Gollwitzer et al., 2018). None of these six studies reported sufficient effect sizes (0.16–0.26). For example, Internet cognitive behavioral therapy (iCBT) (Imamura et al., 2015), Internet-based stress management programs using problem-solving and emotion-regulation (Ebert et al., 2016a; Heber et al., 2016), and a web-based stress and depression literacy interventions were reported to significantly improve work engagement (Imamura et al., 2017); however, their effect sizes were small (0.16–0.26). In sum, previous intervention studies have generally reported a small effect size for work engagement. A more effective intervention should be developed and tested.

One possible reason why these previous studies failed to find large intervention effects on work engagement is that these studies did not employ sufficient strategies to simultaneously improve two important antecedents of work engagement, specifically, job resources and personal resources (Bakker and Demerouti, 2007, 2017; Halbesleben, 2010; Christian et al., 2011). Job resources are defined as physical, social, or organizational aspects of the job that facilitate the achievement of working goals; stimulate personal growth, learning, and development; or reduce job demands or associated physical or psychological costs, such as support from colleagues and opportunity for development (Bakker and Demerouti, 2007). Personal resources are positive self-evaluations linked to resiliency, such as optimism and self-efficacy, and which refer to individuals’ perceptions of their ability to control and have an effect on their environment (Hobfoll et al., 2003; Bakker, 2011; Bakker and Demerouti, 2017). Specifically, most of the previous intervention studies to improve work engagement have employed programs that focused only on personal resources through cognitive behavioral approaches (Imamura et al., 2015, 2017; Bernburg et al., 2016; Ebert et al., 2016a, b; Heber et al., 2016), enhancing career management skills (Vuori et al., 2012), mindfulness training sessions (van Berkel et al., 2014; van Dongen et al., 2016; Klatt et al., 2017; Steinberg et al., 2017), or a self-regulation technique training (Gollwitzer et al., 2018). Only a few interventions focused on job resources, such as changing the physical and social work environment (Coffeng et al., 2014), implementing manager training (Ângelo and Chambel, 2013), or training to reduce physical workload and improve social work environment (Hengel et al., 2012). Other interventions used neither job nor personal resources; instead, they incorporated short-time exercise regimens including stretching or aerobic exercises (Michishita et al., 2017) or lifestyle changes such as fitness (Strijk et al., 2013). An intervention aimed at simultaneously improving both job and personal resources might have a better intervention effect.

### Job Crafting

Job crafting, which refers to employee-initiated design/redesign of work characteristics, could be an effective approach to improve both job and personal resources (Wrzesniewski and Dutton, 2001; Bakker, 2011; Bakker and Demerouti, 2017). Wrzesniewski and Dutton defined job crafting as “the physical and cognitive change individuals make in the task or relational boundaries of their work” (Wrzesniewski and Dutton, 2001). It consists of three components: changing the job’s boundaries (task crafting), changing the relational boundaries (relational crafting), and changing the cognitive task boundaries (cognitive crafting) (Wrzesniewski and Dutton, 2001; Sekiguchi et al., 2014). Task crafting and relational crafting are associated with designing and improving one’s work and social environment in the workplace, which could increase job resources such as job autonomy or social support (Wrzesniewski and Dutton, 2001; Demerouti, 2014). In a longitudinal study, Tims et al. (2013) reported that job crafting was related to job resources, which were job autonomy and social support. Next, cognitive crafting, such as reframing one’s job as significant and meaningful or redefining the purpose and meaning of the job (Wrzesniewski and Dutton, 2001; Sekiguchi et al., 2014), can be related to personal resources, such as optimism and self-efficacy (Bakker, 2011; Bakker and Demerouti, 2017).

According to a meta-analysis of observational studies, job crafting is positively associated with work engagement as well as other health- and job-related outcomes, such as work performance and (low levels of) burnout and emotional exhaustion (Rudolph et al., 2017). In one longitudinal study, job crafting was positively associated with personal resources, such as psychological capital (hope, resilience, self-efficacy, and optimism) (Vogt et al., 2016), which was also associated with work engagement (Wang et al., 2017). Job crafting, as a technique to enhance both job and personal resources of workers, may be a promising intervention to improve work engagement with a larger effect size.

Nevertheless, while eight non-RCTs (van den Heuvel et al., 2015; Dubbelt et al., 2016; Demerouti et al., 2017; Kooij et al., 2017; Van Wingerden et al., 2017a,b,c; Gordon et al., 2018) and one pretest-posttest study (Sakuraya et al., 2016) have been conducted, no previous RCT has explored the effect of a job crafting intervention program on work engagement or other work-related outcomes. Most of these studies used a one-to-five group session format in which participants received training on job crafting, and their task was to create an individual job crafting plan (van den Heuvel et al., 2015; Dubbelt et al., 2016; Sakuraya et al., 2016; Demerouti et al., 2017; Van Wingerden et al., 2017a,b,c; Gordon et al., 2018). Among these eight studies, six examined the effect of the job crafting intervention on work engagement (Dubbelt et al., 2016; Sakuraya et al., 2016; Van Wingerden et al., 2017a,b,c; Gordon et al., 2018); four of the six showed a significant effect on increasing work engagement (Dubbelt et al., 2016; Sakuraya et al., 2016; Van Wingerden et al., 2017a; Gordon et al., 2018), while two found no significant effect (Van Wingerden et al., 2017b,c). However, in four of the studies, effect sizes of work engagement were small (0.05–0.36), as calculated by the first author and as shown in tables in the articles (Dubbelt et al., 2016; Sakuraya et al., 2016; Van Wingerden et al., 2017b,c). The main reason for this conflicting result may be that RCT study design was not used (van den Heuvel et al., 2015; Dubbelt et al., 2016; Sakuraya et al., 2016; Demerouti et al., 2017; Kooij et al., 2017; Van Wingerden et al., 2017a,b,c; Gordon et al., 2018), which could cause biased results. In addition, the participants’ characteristics, such as occupation, varied, which may also account for the conflicting findings. Notwithstanding inconsistent results, previous studies have generally supported the effectiveness of a job crafting intervention on work engagement. The effectiveness of job crafting interventions should be tested and confirmed with a RCT to accumulate a higher quality of evidence.

Additionally, job crafting may have a ceiling effect. For instance, employees having high levels of job crafting at baseline would have already experienced various job crafting behaviors before; thus, it would be difficult for them to increase job crafting more. Thus, we hypothesized that employees with low levels of job crafting at baseline, who conducted less job crafting behavior, may be more engaged in crafting their jobs after the intervention, which would allow more room for improvement in work engagement (Petrou et al., 2015; Tims et al., 2015; Rudolph et al., 2017; Sakuraya et al., 2017). However, in contrast to this hypothesis, Dubbelt et al. (2016) suggested that the participants’ past experiences with job crafting could play an essential role in the learning process and facilitate actual behavioral change, thus enhancing competence in job crafting (Kolb et al., 2001; Dubbelt et al., 2016). This would indicate that workers with low job crafting scores at baseline and less job crafting experiences could have difficulty increasing job crafting after the intervention. Therefore, whether job crafting at baseline would affect the outcomes of the intervention positively or negatively should be investigated.

## Objectives

Accordingly, the aim of the current study was to investigate the effect of job crafting intervention on work engagement among Japanese employees at 3- and 6-month follow-up using a RCT design. As a secondary outcome, the effect on job crafting was also examined. In addition, the effects on the outcomes were investigated separately among high and low score of job crafting.

## Methods

### Trial Design

This study design was a RCT. The allocation ratio of the intervention group to the control group was 1:1. The Research Ethics Review Board of Graduate School of Medicine/Faculty of Medicine, the University of Tokyo approved the study procedures (reference number: 10749). The protocol was registered at the UMIN Clinical Trials Registry (UMIN-CTR) (ID=UMIN000026668). The manuscript was written according to the Consolidated Standards of Reporting Trials (CONSORT) checklist (Schulz et al., 2010) (Supplementary Appendix S1).

### Participants

Six workplaces (five private companies A-E, and one public elementary school F) participated in the current study. Specifically, companies A and D were in the service industry and, companies B, C, and E were in the manufacturing industry. All of them were in the Tokyo area. All workers in company A (*N* = 1,812), B (*N* = 1,328), C (*N* = 1,914), D (head office: *n* = 200, Branch office: *n* = 20), and E (head office: *n* = 45, Branch office: *n* = 26), and all workers in elementary school F (*N* = 58) were recruited by a contact person in their own company or elementary school using an invitation e-mail or letter. Inclusion criteria for participants were: (1) currently employed, and (2) could participate in the intervention (two workshops). No exclusion criteria were specified in the current study. In the five companies A-E, those who were interested in participating in this study were asked to access a research website to review the study’s objectives and procedures and to answer the web-based self-report questionnaire in Japanese at baseline if they agreed to participate. In elementary school F, the participants read the same explanation of the study and completed a paper form of the self-report questionnaire at baseline because they did not have their own PCs in their workplace.

### Interventions

The original-version of the job crafting intervention program was previously developed and examined in a pretest-posttest study by the first author and colleagues, which reported a significant effect of improving work engagement and psychological distress (Sakuraya et al., 2016). The strong point of the program was that it focused on three types of job crafting (i.e., task, relation, and cognition) based on the concept of Wrzesniewski and Dutton (2001), which could be useful in improving work engagement. In the current study, we modified the original-version of the program (Wrzesniewski and Dutton, 2001). The modified version consisted of two 120-minute job crafting sessions conducted by first author at monthly intervals. From the participants’ opinions collected through the pretest-posttest study and discussion with occupational health professionals, two improvements were made. First, job crafting cases were collected in a booklet and distributed to the participants during the first session so that they could learn various job crafting cases more easily, which would also help them make their job crafting plan more reasonable. Second, e-mail or letter follow-up was added after the first and second session, which aimed to help the participants more easily remember the session and daily practice their job crafting plan. After each session, the first author sent an e-mail or letter that reflected the contents of the session and work to review their job crafting plan. If the participants had any question about job crafting, they could ask the first author at any time during the intervention period, which was from the first session to 1 month after the second session. The participants who could not attend the first or second session were given the material from the session and asked to create their job crafting plan and conduct it. No incentive was offered for participating in the program.

### Control Group Conditions

The participants in the control group received no intervention from baseline to the 6-month follow-up survey. After the 6-month follow-up, the first author administered the same job crafting intervention program.

### Outcomes

All data were collected using a web-based self-report questionnaire in companies A-E or a paper-based questionnaire in elementary school F at baseline, 3-month, and 6-month follow-up after baseline survey. Intervention started approximately 1 month after baseline survey.

### Primary Outcome

#### Work Engagement

Work engagement was measured using the Japanese version of the Utrecht Work Engagement Scale (UWES), which has been reported to be reliable and valid (Shimazu et al., 2008). It comprises 9 items, with 3 items assessing vigor (e.g., “At my work, I feel bursting with energy.”), 3 items assessing dedication (e.g., “My job inspires me.”), and 3 items assessing absorption (e.g., “I get carried away when I am working.”). All items were rated on a 7-point Likert scale ranging from 0 (never) to 6 (always), and the total score was divided by the number of items to obtain an average score. Shimazu et al. (2008) reported reliability of the scale by Cronbach’s α of 0.92, and the factorial validity by confirmatory factor analysis (CFA) of this full scale (χ2 [97] = 1067.07; GFI = 0.90; AGFI = 0.86; RMSEA = 0.07; CFI = 0.92) (59).

### Secondary Outcome

#### Job Crafting

Job crafting was measured using a scale developed by Sekiguchi et al. (2014) based on Wrzesniewski and Dutton’s (2001) conceptualization of the concept (Wrzesniewski and Dutton, 2001), which has been reported to be reliable and valid. It comprises 12 items assessing three subscales: task crafting (4 items; e.g., “Add or reduce tasks so that my job can be performed more smoothly”), relational crafting (4 items; e.g., “Actively interact with people through my job”), and cognitive crafting (4 items; e.g., “Reframe my job as significant and meaningful”). All items were measured on a 7-point Likert scale ranging from 1 (strongly disagree) to 7 (strongly agree). The total score, as well as each subscale score, was calculated by dividing the sum of item scores by the number of the items. Sekiguchi et al. (2014) supported the reliability of the scale, reporting the Cronbach’s α of 0.78 for this full scale (Sekiguchi et al., 2014). The results of CFA supported the three-dimensional structure of this scale (χ2[17] = 115.76; RMSEA = 0.11; CFI = 0.95; NNFI = 0.92) (Sekiguchi et al., 2014).

### Demographic Characteristics

Demographic characteristics included age, gender, marital status, occupation, education, and employment.

### Sample Size

The estimated sample size necessary to detect an effect size (Cohen’s *d*) of 0.3 or greater for work engagement at an alpha error rate of 0.05 (two-tailed) and a beta error rate of 0.20 using the G* Power 3 program was 352 participants (176 participants per arm) (Faul et al., 2007, 2009).

There have been no previous RCT studies reporting effect sizes of job crafting intervention programs on work engagement. However, a meta-analysis reported the effect size (Cohen’s *d*) of 0.29 for any intervention on work engagement (Knight et al., 2017). In addition, in a previous pretest-posttest study on job crafting conducted by the first author, the effect size was about 0.3 (Sakuraya et al., 2016). Thus, it seemed reasonable to set 0.3 as an expected effect size in the current job crafting intervention program.

### Randomization

Participants who fulfilled the inclusion criteria and completed a questionnaire at baseline were randomly allocated to the intervention or the control groups. Stratified permuted-block randomization was conducted. Participants were stratified into eight strata according to the workplace to which they belonged (company A or B or C, or head office or branch office of company D or E, respectively, or elementary school F). An independent researcher generated a stratified permuted-block random table. An independent research assistant conducted enrolment and assignment. The stratified permuted-block random table, which was password-protected, was blinded to the authors. Only the research assistant had access to it during the process of random allocation of participants.

### Statistical Methods

A mixed-model for repeated measures conditional growth model analysis was conducted using a group (intervention and control) × time (baseline, 3-month, and 6-month follow-up) interaction as an indicator of intervention effect. Intention-to-treat analysis (ITT) was used. First, several mixed models were applied to the data: random intercept and random slope; random intercept only; and random slope only. A converged model that showed the smallest AIC (Akaike Information Criterion), an indicator of goodness of fit of the model, was selected. If these mixed models did not converge, a fixed model was used. As a sensitivity analysis, a mixed model for repeated measures analysis of variance model analysis was conducted. The linear mixed model in SPSS Statistics 25.0 (SPSS Inc., Chicago, IL, United States) was used. Additionally, the effect sizes (95% CIs) were calculated using Cohen’s *d* only among those who completed the questionnaire at 3- and 6-month follow-up, although the effect sizes may be biased due to drop-outs. Values of 0.2, 0.5, and 0.8 are generally interpreted as small, medium, and large effects, respectively, Cohen (1992). Subgroup analyses were conducted separately for respondents who had high scores (higher than 5) and low scores (5 or lower score) of job crafting at baseline. Because there was no standard cut off point of job crafting, the median score was used as alternatively for keeping homogeneity of the sample size of each subgroup. Thus, in the current study, employees with low levels of job crafting at baseline means those having 5 or lower score of job crafting at that time.

### Change to Protocol

Some changes made to the protocol registered at the UMIN Clinical Trials Registry (UMIN-CTR) (ID=UMIN000026668) are the inclusion criteria and subgroup analysis. Originally, it was planned that only the participants who were regular employees would be allocated. Before the commencement of this study, this criterion was canceled due to the anticipation of a low participation rate. Additionally, subgroup analyses were conducted separately for respondents who had high scores (higher than 5) and low scores (5 or lower score) of job crafting at baseline, which was not planned before.

### Patient and Public Involvement

The current job crafting intervention program was developed based on interviews with employees on how they craft their own job in their working lives. Before starting this study, contact persons in participating companies and the elementary school reviewed and commented on the intervention program based on their priorities and experiences. They also helped recruiting and conducting the study. The results of the study were reported to all participating companies and the elementary school.

## Results

### Participant Recruitment

**FIGURE 1:**
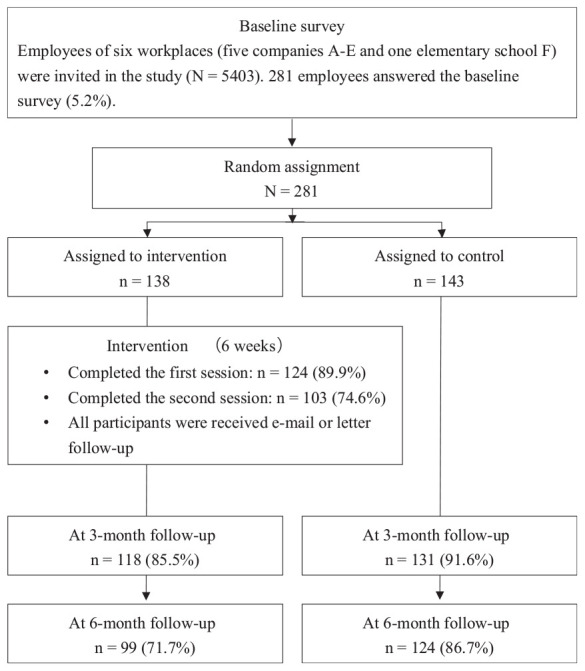
Participant flowchart.

Recruitment and the baseline survey were conducted from April to May 2017. The intervention and control groups were assessed at approximately 3 months (from July to August 2017) and 6 months (from October to November 2017) after the baseline survey. Figure 1 shows the participant flowchart. Participants were collected from six workplaces (five companies A-E and one elementary school F) (*N* = 5,403), and 281 (5.2%) of them completed a baseline survey. All of them were randomly allocated to an intervention group (*n* = 138) or control group (*n* = 143). At 3-month follow-up, 118 (85.5%) participants in the intervention group and 131 (91.6%) in the control group completed the survey. At 6-month follow-up, 99 (71.7%) participants in the intervention group and 124 (86.7%) in the control group completed the survey. At each follow-up survey, the response rate of the intervention group was lower compared to that of the control group. Reasons for dropping out were not assessed in this study.

### Baseline Data

Table 1 presents demographic characteristics. In both groups, most participants were male (Intervention: 59.4%, Control: 60.8%), and most participants completed university or higher education (Intervention: 82.6%, Control: 79.0%) and held regular employment (Intervention: 84.8%, Control: 83.9%). Most frequent occupations were professionals (Intervention: 50.7%, Control: 41.3%), in addition, sales, clerical, and managers.

**TABLE 1:** Baseline characteristics of participants in the intervention and control groups (*N* = 281).

	Intervention group (*n* = 138)				Control group (*n* = 143)			
	*n*	%	Mean	SD	*n*	%	Mean	SD
Age			35.65	8.34			37.49	9.05
**Sex**								
Male	82	59.4			87	60.8		
Female	56	40.6			56	39.2		
**Marital status**								
Not married	64	46.4			72	50.4		
Married	74	53.6			71	49.7		
**Occupation**								
Manager	14	10.1			20	14.0		
Professional	70	50.7			59	41.3		
Clerical	19	13.8			19	13.3		
Physical work (blue-collar)	1	0.7			2	1.4		
Sales	23	16.7			27	18.9		
Others	11	8.0			16	11.2		
**Education**								
High school or some college	24	17.4			30	21.0		
University or higher	114	82.6			113	79.0		
**Employment**								
Regular	117	84.8			120	83.9		
Non-regular*	21	15.2			23	16.1		

* Temporary staff, contact employee, and part-time employee.

### Effects of the Job Crafting Intervention Program on Each Outcome Variable

Table 2 presents the means and SDs of the outcome variables at baseline, 3-month, and 6-month follow-up in the intervention and the control groups. Table 3 shows the estimated effects of the job crafting intervention program on the outcome variables based on the mixed model analyses as well as effect sizes (Cohen’s *d*). None of the growth models including random effects converged; thus, only fixed effect results from the model including are reported here. Regarding the variance model, the model that included random intercept was adopted. The job crafting intervention program showed a non-significant effect on work engagement. The effect sizes for work engagement were small, with values of 0.15 (95% CI: −0.10 to 0.40) at 3-month follow-up and 0.03 (95% CI: −0.24 to 0.29) at 6-month follow-up. The job crafting intervention program had a non-significant effect on job crafting, which effect size was also small.

**TABLE 2:** Means (SDs) of outcome variables at baseline, 3-, and 6-month follow-up in the intervention and control groups for the whole sample.

		Intervention								
		Baseline			3-month			6-month		
	Range	n*	Mean	SD	n*	Mean	SD	n*	Mean	SD
Work engagement	0–6	138	3.01	1.06	118	3.03	1.09	99	2.80	1.06
Job crafting	1–7	137	5.00	0.89	118	5.08	0.94	99	5.01	0.88
Task crafting	1–7	137	5.24	0.91	118	5.32	0.95	99	5.25	0.82
Relational crafting	1–7	137	4.93	1.07	118	4.99	1.09	99	4.99	1.03
Cognitive crafting	1–7	137	4.83	1.28	118	4.94	1.28	99	4.77	1.22

		**Control**								
	
		**Baseline**			**3-month**			**6-month**		
				
	**Range**	n*	**Mean**	**SD**	n*	**Mean**	**SD**	n*	**Mean**	**SD**

Work engagement	0–6	143	3.21	1.16	131	3.11	1.25	124	2.94	1.19
Job crafting	1–7	142	5.00	0.93	130	4.99	0.94	124	4.89	0.96
Task crafting	1–7	142	5.22	0.99	131	5.23	0.99	124	5.08	1.00
Relational crafting	1–7	142	4.94	1.11	130	4.95	1.07	124	4.90	1.06
Cognitive crafting	1–7	142	4.84	1.26	131	4.79	1.34	124	4.71	1.30

* Because of missing values, the number of respondents for some variables were small.

**TABLE 3:** Effects of the job crafting intervention program on work-related outcomes variables for the whole sample (*N* = 281).

	Int (*n* = 138)		Cont (*n* = 143)			95% CI of estimates of fixed efects			
	EM	SE	EM	SE	Estimates of fixed effects	Lower	Higher	t	*p*
**Work engagement**									
3-month	3.02	0.10	3.12	0.10	0.10	−0.07	0.26	1.11	0.27
6-month	2.82	0.10	2.98	0.10	0.04	−0.15	0.22	0.38	0.71
Pooled					0.03	−0.09	0.15	0.52	0.60
Job crafting*									
3-month	5.09	0.08	5.00	0.08	0.09	−0.09	0.27	0.97	0.33
6-month	5.00	0.09	4.91	0.08	0.09	−0.12	0.29	0.81	0.42
Pooled					0.05	−0.07	0.17	0.90	0.37
Task crafting*									
3-month	5.31	0.09	5.25	0.08	0.05	−0.16	0.25	0.44	0.66
6-month	5.20	0.09	5.09	0.08	0.10	−0.13	0.32	0.87	0.39
Pooled					0.06	−0.07	0.20	0.96	0.34
Relational crafting*									
3-month	5.00	0.10	4.96	0.09	0.05	−0.19	0.29	0.44	0.66
6-month	4.97	0.10	4.92	0.09	0.06	−0.21	0.34	0.45	0.66
Pooled					0.04	−0.11	0.19	0.52	0.60
Cognitive crafting*									
3-month	4.95	0.11	4.79	0.11	0.17	−0.10	0.44	1.27	0.21
6-month	4.82	0.12	4.73	0.11	0.10	−0.18	0.38	0.69	0.49
Pooled					0.06	−0.11	0.24	0.69	0.49

			95% CI of Cohen’s *d*	
			
	*N*	Cohen’s *d*	Lower	Higher
**Work engagement**				
3-month**	249	0.15	−0.10	0.40
6-month***	223	0.03	−0.24	0.29
**Job crafting**				
3-month**	247	0.12	−0.13	0.37
6-month***	222	0.06	−0.21	0.32
**Task crafting**				
3-month**	248	0.05	−0.20	0.30
6-month***	222	0.05	−0.22	0.31
**Relational crafting**				
3-month**	247	0.06	−0.19	0.31
6-month***	222	0.01	−0.26	0.27
**Cognitive crafting**				
3-month**	248	0.17	−0.08	0.42
6-month***	222	0.08	−0.18	0.35

Int = Intervention, Cont = Control, EM = Estimated means, SE = Standard errors. * N = 280, which was because of one missing value at any of the surveys (baseline, 3-months, or 6-moths follow-up). ** Cohen’s d between baseline and 3-month follow-up survey were based on the score of the respondents who completed both surveys. *** Cohen’s d between baseline and 6-month follow-up survey were based on the score of the respondents who completed both surveys.

### Subgroup Analyses

Tables 4a,b show the estimated intervention effects of the job crafting intervention program on the outcomes based on the mixed-model analyses conducted separately for subgroups. In the lower job crafting subgroup (Table 4a), a significant effect on work engagement at 3-month follow-up (*t* = 2.02, *p* = 0.04) was shown, although the effect size was small and not significant (*d* = 0.33, 95% CI: −0.004 to 0.67).

**TABLE 4a:** Effects of the job crafting intervention program on work-related outcomes for lower job crafting (job crafting scale ≤ 5.00) (*n* = 152).

	Int (*n* = 70)		Cont (*n* = 82)			95% CI			
	EM	SE	EM	SE	Estimates of fixed effects	Lower	Higher	t	*p*
**Work engagement**									
3-month	2.59	0.12	2.61	0.11	0.20	0.01	0.40	2.02	0.04
6-month	2.33	0.13	2.52	0.11	0.03	−0.22	0.29	0.26	0.80
Pooled					0.04	−0.10	0.18	0.54	0.59
**Job crafting**									
3-month	4.58	0.09	4.54	0.08	0.08	−0.16	0.32	0.66	0.51
6-month	4.55	0.10	4.43	0.08	0.15	−0.13	0.44	1.08	0.28
Pooled					0.08	−0.07	0.24	1.09	0.28
**Task crafting**									
3-month	4.84	0.11	4.89	0.09	−0.02	−0.31	0.27	−0.12	0.91
6-month	4.88	0.12	4.66	0.10	0.25	−0.09	0.58	1.47	0.14
Pooled					0.13	−0.05	0.31	1.42	0.16
**Relational crafting**									
3-month	4.47	0.12	4.49	0.11	0.04	−0.30	0.39	0.25	0.80
6-month	4.52	0.14	4.45	0.11	0.13	−0.27	0.53	0.66	0.51
Pooled					0.07	−0.14	0.28	0.67	0.50
**Cognitive crafting**									
3-month	4.43	0.15	4.24	0.13	0.22	−0.14	0.57	1.21	0.23
6-month	4.29	0.16	4.19	0.13	0.12	−0.25	0.50	0.66	0.51
Pooled					0.06	−0.17	0.29	0.48	0.64

						**95% CI**			
		***n***		**Cohen’s *d***		**Lower**			**Higher**

**Work engagement**				
3-month *		134		0.33		−0.004			0.67
6-month **		120		0.02		−0.35			0.39
**Job crafting**				
3-month *		134		0.12		−0.23			0.46
6-month **		120		0.25		−0.12			0.61
**Task crafting**				
3-month *		134		−0.02		−0.36			0.32
6-month **		120		0.22		−0.15			0.58
**Relational crafting**				
3-month *		134		0.06		−0.28			0.41
6-month **		120		0.12		−0.25			0.49
**Cognitive crafting**				
3-month *		134		0.20		−0.14			0.54
6-month **		120		0.22		−0.15			0.58

Int, Intervention; Cont, Control; EM, Estimated means; SE, Standard errors.* n = 151, which was because of one missing value at baseline survey. * Cohen’s d between baseline and 3-month follow-up survey were based on the score of the respondents who completed both surveys. ** Cohen’s d between baseline and 6-month follow-up survey were based on the score of the respondents who completed both surveys.

**TABLE 4b:** Effects of the job crafting intervention program on work-related outcomes for higher job crafting (job crafting scale > 5.00) (*n* = 127).

	Int (*n* = 67)		Cont (*n* = 60)			95% CI of estimates of fixed efects			
	EM	SE	EM	SE	Estimates of fixed effects	Lower	Higher	t	*p*
**Work engagement**									
3-month	3.45	0.13	3.81	0.14	−0.04	−0.33	0.25	−0.27	0.79
6-month	3.31	0.13	3.60	0.14	0.03	−0.26	0.31	0.19	0.85
Pooled					0.01	−0.18	0.20	0.09	0.93
**Job crafting**									
3-month	5.62	0.09	5.63	0.09	0.15	−0.11	0.41	1.17	0.25
6-month	5.44	0.09	5.58	0.09	0.02	−0.28	0.33	0.15	0.88
Pooled					0.02	−0.14	0.18	0.24	0.81
**Task crafting**									
3-month	5.80	0.10	5.73	0.10	0.18	−0.09	0.44	1.32	0.19
6-month	5.55	0.10	5.68	0.10	−0.01	−0.31	0.28	−0.09	0.93
Pooled					0.005	−0.16	0.17	0.05	0.96
**Relational crafting**									
3-month	5.54	0.11	5.60	0.11	0.10	−0.23	0.42	0.58	0.56
6-month	5.41	0.11	5.58	0.11	−0.02	−0.39	0.36	−0.08	0.94
Pooled					−0.002	−0.20	0.19	−0.02	0.98
**Cognitive crafting**									
3-month	5.50	0.13	5.55	0.13	0.19	−0.20	0.58	0.98	0.33
6-month	5.34	0.13	5.48	0.14	0.09	−0.34	0.52	0.43	0.67
Pooled					0.05	−0.18	0.29	0.45	0.65

			95% CI of Cohen’s *d*	
			
	*n*	Cohen’s *d*	Lower	Higher
**Work engagement**				
3-month*	114	0.01	−0.36	0.37
6-month**	102	0.04	−0.35	0.43
**Job crafting**				
3-month*	113	0.24	−0.13	0.61
6-month**	102	0.01	−0.38	0.40
**Task crafting**				
3-month*	114	0.22	−0.15	0.58
6-month**	102	−0.04	−0.43	0.35
**Relational crafting**				
3-month*	113	0.14	−0.23	0.51
6-month**	102	−0.03	−0.42	0.36
**Cognitive crafting**				
3-month*	114	0.22	−0.14	0.59
6-month**	102	0.08	−0.31	0.47

*Int, Intervention; Cont, Control; EM, Estimated means; SE, Standard errors.*

* Cohen’s d between baseline and 3-month follow-up survey were based on the score of the respondents who completed both surveys.

** Cohen’s d between baseline and 6-month follow-up survey were based on the score of the respondents who completed both surveys.

### Process Evaluation, Satisfaction With, and Understanding of Each Job Crafting Session

In the intervention group, 124 (89.9%) completed the first session, 103 (74.6%) completed the second session. Table 5 shows the degree of satisfaction with and understanding of each job crafting session among participants of the intervention group (*n* = 118 and 99 in the first and second session, respectively). Most participants (over 80%) were satisfied with each session and could understand the contents.

**TABLE 5:** The degree of satisfaction and understanding for each job crafting session among participants of the intervention group (*n* = 118 and 99 in the first and second session, respectively).

	First session (*n* = 118)*		Second session (*n* = 99)**	
Item	*n*	%	*n*	%
**Satisfaction**				
Satisfied	55	46.6	49	49.5
A little satisfied	46	39.0	38	38.4
Neither	15	12.7	9	9.1
A little dissatisfied	2	1.7	2	2.0
Dissatisfied	–	–	1	1.0
**Understanding**				
Understood	101	85.6	89	89.9
Neither	14	11.9	10	10.1
was difficult	3	2.5	–	–

– no case. * Response rate was 95.2%. ** Response rate was 96.1%.

## Discussion

The current study examined the effects of a newly developed job crafting intervention program on improving work engagement and other outcomes at 3-month and 6-month follow-up among workers in Japan. In the total sample, the job crafting intervention program had a non-significant effect on work engagement at both 3-month and 6-month follow-up. In addition, job crafting did not improve significantly in the intervention group compared to the control group. However, the program had significant intervention effects on work engagement for the lower job crafting subgroup. The job crafting intervention program may not be sufficiently effective in improving work engagement and job crafting for the entire sample. However, it may be effective for workers who have lower job crafting.

The job crafting intervention program did not significantly improve work engagement or job crafting in the intervention group compared to the control group. These results are inconsistent with previous studies, which showed significant effects of job crafting intervention on improving work engagement (Dubbelt et al., 2016; Sakuraya et al., 2016; Van Wingerden et al., 2017a; Gordon et al., 2018) and job crafting (Dubbelt et al., 2016; Sakuraya et al., 2016; Van Wingerden et al., 2017a) in a non-RCT and a pretest-posttest study. One possible reason may be the number of group sessions. For example, Van Wingerden et al. (2017a, b,c) and Gordon et al. (2018) administered three and four sessions, respectively (Van Wingerden et al., 2017a; Gordon et al., 2018), whereas the current study administered only two. Although participants in the current study received an e-mail or a letter follow-up after the first and second sessions, these interactions could be less intensive compared to a face-to-face session. Second, not having participants reflect on past job crafting experiences—which could support participants in learning and increase more job crafting behavior—may have decreased the effect of the current intervention. In the program reported by Dubbelt et al. (2016), participants reflected upon their past experiences with job crafting before creating new job crafting plans, which could enhance their job crafting behavior (Dubbelt et al., 2016). Based on experiential learning theory, participants’ past experiences with job crafting can play an important role in the learning process and facilitate actual behavior (Kolb et al., 2001). In the current study, such self-reflection exercise was not included, which might have decreased the effectiveness of the current program on increasing job crafting. For further study, we should provide simpler and more reasonable job crafting cases for participants so that they could more easily benefit from the examples of various job crafting cases even if they had few job crafting experiences of their own. Furthermore, e-mail follow-ups should be conducted more frequently after each session, which encourage them to try their job crafting plan. Third, in the current study, the participation rate in the second session (74.6%) was lower than the 84.0% reported in Sakuraya et al. (2016). Although participants who could not attend the session received the materials from the session and were asked to create their job crafting plan, the low participation rate would weaken the effect of the intervention. For these reasons, the current job crafting intervention program could not effectively increase job crafting behavior, which may have contributed to its insignificant effects on improving work engagement.

However, for workers with low job crafting, work engagement was found to increase significantly at 3-month follow-up in the intervention group compared with the control group (*p* = 0.04), although the effect size was small and non-significant (Cohen’s *d* = 0.33). This result could be because they were motivated by the group session, in which they reflected on their recent work style and discussed how they could work more positively among other members. This opportunity might have helped them appreciate the present and view the future as an opportunity or receive positive feedback from other participants, which could improve their personal resources, such as optimism and self-efficacy (Schneider, 2001; Luthans et al., 2008; Wingerden et al., 2016) as predictors of work engagement (Halbesleben, 2010; Bakker, 2011; Bakker and Demerouti, 2017). For workers with low job crafting, reflecting upon their work style and thinking about the ways to work more positively may be new experience; thus, such group sessions could be more interesting for them. Accordingly, for workers with low job crafting, the current program could effectively increase work engagement in the intervention group compared with the control group.

In this study, 124 (89.9%) participants completed the first session and 103 (74.6%) completed the second session, which was lower compared to previous job crafting intervention studies (Sakuraya et al., 2016). As mentioned above, the low rates of completing the sessions may have decreased the effect of the intervention in this study. Hence, completion rates would have to be higher to improve the effect of the job crafting intervention program. Next, most participants who experienced the intervention program (over 80%) were satisfied with each session, and they could understand the contents, supporting the content validity of this job crafting intervention program.

## Limitations

The current study had several limitations. First, this study did not utilize a stratified permuted-block randomization into lower or higher levels of job crafting subgroups at baseline. Instead, participants were separated into two groups by a simple randomization, which could have led to biased assignment of the participants into the intervention and the control groups. Second, participants were recruited from six worksites (five companies and one elementary school) in Japan. Most participants had higher education levels, which might have helped them learn the contents of the job crafting intervention program more easily. Therefore, generalization of the present findings to the working population is limited. Third, the sample size of this study (*N* = 281) was modest compared to the estimated number of 352 needed to detect an effect size of 0.3 or greater for work engagement. Thus, the study had lower statistical power. Fourth, only 5% of the participants submitted their optional homework, which asked them to complete a reflection sheet describing their job crafting plan. This percentage was much lower compared to 24.4% reported in a previous RCT study in which internet-based cognitive behavioral therapy intervention significantly improved work engagement (Imamura et al., 2015). This low rate of submitting homework may have weakened the effect of the intervention. Future job crafting intervention programs should encourage the participants to submit homework by using e-mail reminder messages or the use of mobile devices. Fifth, dropout rates at 6-month follow-up were 28.3% in the intervention group and 13.3% in the control group. These were similar to those of previous RCT study in which the intervention significantly increased work engagement (28.6 and 16%, respectively) (Imamura et al., 2015). Nevertheless, the dropouts might have led to a dropout bias, particularly if the intervention group participants with low levels of work engagement were more likely to quit the follow-up survey. Sixth, participants in the control group could get information about the job crafting intervention program from participants in the intervention group, since they worked in the same workplace. Such a contamination may weaken the intervention effect. Finally, all outcomes in the current study were assessed by self-report, which could have been affected by participants’ perceptions or situational factors related to work. A self-reported measure could be vulnerable to a cognitive bias. A future study should consider the use of objectively measured outcomes.

### Theoretical Implication

To our knowledge, this is the first study to investigate the effect of the job crafting intervention program on work engagement and job crafting in RCT design. Previous studies were non-RCT, yielding controversial effects of job crafting on work engagement. In the current study, for the whole sample, the effects of the job crafting intervention program on work engagement and job crafting were not significant. However, the current job crafting intervention program showed improved work engagement of workers low on job crafting in the intervention group compared to the control group. This may suggest that the intervention might be more effective in increasing work engagement of workers who had less conducted job crafting in the past. On the other hand, workers high on job crafting may need to undergo a more intensive job crafting intervention program that would include advanced examples of job crafting that they had not tried before. Thus, the job crafting intervention program would be more useful if it were designed according to job crafting score at baseline.

### Practical Implication

The current findings showed that the job crafting intervention program composed of task, relational, and cognitive crafting could effectively increase work engagement of workers low on job crafting. This job crafting intervention program may be used as a new strategy to improve well-being of workers who had done less job crafting. Additionally, it may imply that job crafting strategy would differ according to the job crafting behavior at baseline; hence, future studies should tailor job crafting intervention program accordingly. For example, for workers who had done less job crafting, providing simpler and more reasonable job crafting cases would be useful so that they could more easily benefit from the examples of various job crafting cases even if they had few job crafting experiences of their own. For workers who had conducted more job crafting already, more intensive program would be useful, such as providing more advanced examples of job crafting and supporting them trying more job crafting behavior. Based on these points, we have to improve the job crafting program to be more effective for all workers.

## Conclusion

This study first examined the effect of job crafting intervention on work engagement and job crafting among Japanese employees in a randomized controlled study. The program effectively increased work engagement of workers lower on job crafting.

## Data Availability Statement

The raw data supporting the conclusions of this article will be made available by the authors, without undue reservation, to any qualified researcher.

## Ethics Statement

The Research Ethics Review Board of Graduate School of Medicine/Faculty of Medicine, the University of Tokyo approved the study procedures (reference number: 10749). In the five companies A-E, those who were interested in participating in this study were asked to access a research website to review the study’s objectives and procedures and to answer the web-based self- report questionnaire at baseline if they agreed to participate. In elementary school F, the participants read the same explanation of the study and completed a paper form of the self-report questionnaire at baseline because they did not have their own PCs in their workplace.

## Author Contributions

All authors conceived of the study, developed the study design, conducted the literature search, collected, analyzed, and interpreted data, prepared the first draft, reviewed the manuscript and read and approved the final manuscript.

## Conflict of Interest

The authors declare that the research was conducted in the absence of any commercial or financial relationships that could be construed as a potential conflict of interest.

## Supplementary Material

APPENDIX S1 CONSORT checklist 2010.

## Abbreviations

RCT: randomized controlled trial
