# Corrigendum: Effects of a job crafting intervention program on work engagement among Japanese employees: A randomized controlled trial

**DOI:** 10.3389/fpsyg.2023.1153979

**Published:** 2023-08-04

**Authors:** Asuka Sakuraya, Akihito Shimazu, Kotaro Imamura, Norito Kawakami

**Affiliations:** ^1^Department of Public Health, School of Medicine, Tokyo Women's Medical University, Shinjuku, Japan; ^2^Faculty of Policy Management, Keio University, Fujisawa-shi, Japan; ^3^Department of Mental Health, Graduate School of Medicine, The University of Tokyo, Bunkyo, Japan

**Keywords:** job crafting, work engagement, mental health, well-being, employee, randomized controlled trial

In the published article, there were errors in **Tables 2**, **3**, **4b** as published. Due to a miscalculation, a number of values were incorrect.

In **Table 2** changes were made to row Work Engagement at columns Intervention, 3-month, Mean and SD and at columns Intervention, 6-month, Mean and SD.

In **Table 3** changes were made to rows Work Engagement, 3-month, 6-month and Pooled. Changes were also made to column 95% CI of Cohen's d, Lower at row Job Crafting, 6-month. In addition, due to a formatting issue, row Relational Crafting, Pooled has become Task Crafting, Pooled, and row Cognitive Crafting, Pooled has become Relational Crafting, Pooled. In addition the table footnotes have been amended from “^*^ Cohen's d between baseline and 3-month follow-up survey were based on the score of the respondents who completed both surveys. ^**^ Cohen's d between baseline and 6-month follow-up survey were based on the score of the respondents who completed both surveys.” to “^**^ Cohen's d between baseline and 3-month follow-up survey were based on the score of the respondents who completed both surveys. ^***^ Cohen's d between baseline and 6-month follow-up survey were based on the score of the respondents who completed both surveys.” Due to this all instances of “3-month^*^” have become “3-month^**^” and all instances of “6-month^**^” have become “6-month^***^”.

In **Table 4b** changes were made to rows, Work Engagement, 3-month, 6-month and Pooled.

The revised Tables 2, 3, 4b are provided below.

**Table 2:** Means (SDs) of outcome variables at baseline, 3-, and 6-month follow-up in the intervention and control groups for the whole sample.

		**Intervention**								
		**Baseline**			**3-month**			**6-month**		
	**Range**	*n* ^*^	**Mean**	**SD**	*n* ^*^	**Mean**	**SD**	*n* ^*^	**Mean**	**SD**
Work engagement	0–6	138	3.01	1.06	118	3.03	1.09	99	2.80	1.06
Job crafting	1–7	137	5.00	0.89	118	5.08	0.94	99	5.01	0.88
Task crafting	1–7	137	5.24	0.91	118	5.32	0.95	99	5.25	0.82
Relational crafting	1–7	137	4.93	1.07	118	4.99	1.09	99	4.99	1.03
Cognitive crafting	1–7	137	4.83	1.28	118	4.94	1.28	99	4.77	1.22
		**Control**								
		**Baseline**			**3-month**			**6-month**		
	**Range**	*n* ^*^	**Mean**	**SD**	*n* ^*^	**Mean**	**SD**	*n* ^*^	**Mean**	**SD**
Work engagement	0–6	143	3.21	1.16	131	3.11	1.25	124	2.94	1.19
Job crafting	1–7	142	5.00	0.93	130	4.99	0.94	124	4.89	0.96
Task crafting	1–7	142	5.22	0.99	131	5.23	0.99	124	5.08	1.00
Relational crafting	1–7	142	4.94	1.11	130	4.95	1.07	124	4.90	1.06
Cognitive crafting	1–7	142	4.84	1.26	131	4.79	1.34	124	4.71	1.30

* Because of missing values, the number of respondents for some variables were small.

**Table 3:** Effects of the job crafting intervention program on work-related outcomes variables for the whole sample (*N* = 281).

	**Int (*****n*** = **138)**		**Cont (*****n*** = **143)**			**95% CI of estimates of fixed effects**			
	**EM**	**SE**	**EM**	**SE**	**Estimates of fixed effects**	**Lower**	**Higher**	**t**	***p***
**Work engagement**									
3-month	3.02	0.10	3.12	0.10	0.10	−0.07	0.26	1.11	0.27
6-month	2.82	0.10	2.98	0.10	0.04	−0.15	0.22	0.38	0.71
Pooled					0.03	−0.09	0.15	0.52	0.60
**Job crafting** ^*^									
3-month	5.09	0.08	5.00	0.08	0.09	−0.09	0.27	0.97	0.33
6-month	5.00	0.09	4.91	0.08	0.09	−0.12	0.29	0.81	0.42
Pooled					0.05	−0.07	0.17	0.90	0.37
**Task crafting** ^*^									
3-month	5.31	0.09	5.25	0.08	0.05	−0.16	0.25	0.44	0.66
6-month	5.20	0.09	5.09	0.08	0.10	−0.13	0.32	0.87	0.39
Pooled					0.06	−0.07	0.20	0.96	0.34
**Relational crafting** ^*^									
3-month	5.00	0.10	4.96	0.09	0.05	−0.19	0.29	0.44	0.66
6-month	4.97	0.10	4.92	0.09	0.06	−0.21	0.34	0.45	0.66
Pooled					0.04	−0.11	0.19	0.52	0.60
**Cognitive crafting** ^*^									
3-month	4.95	0.11	4.79	0.11	0.17	−0.10	0.44	1.27	0.21
6-month	4.82	0.12	4.73	0.11	0.10	−0.18	0.38	0.69	0.49
Pooled					0.06	−0.11	0.24	0.69	0.49
						**95% CI of Cohen's** ***d***			
	***N***		**Cohen's** ***d***		**Lower**		**Higher**		
**Work engagement**									
3-month^**^	249		0.15		−0.10		0.40		
6-month^***^	223		0.03		−0.24		0.29		
**Job crafting**									
3-month^**^	247		0.12		−0.13		0.37		
6-month^***^	222		0.06		−0.21		0.32		
**Task crafting**									
3-month^**^	248		0.05		−0.20		0.30		
6-month^***^	222		0.05		−0.22		0.31		
**Relational crafting**									
3-month^**^	247		0.06		−0.19		0.31		
6-month^***^	222		0.01		−0.26		0.27		
**Cognitive crafting**									
3-month^**^	248		0.17		−0.08		0.42		
6-month^***^	222		0.08		−0.18		0.35		

Int, Intervention; Cont, Control; EM, Estimated means; SE, Standard errors.

* N = 280, which was because of one missing value at any of the surveys (baseline, 3-month, or 6-month follow-up).

** Cohen's d between baseline and 3-month follow-up survey were based on the score of the respondents who completed both surveys.

*** Cohen's d between baseline and 6-month follow-up survey were based on the score of the respondents who completed both surveys.

**Table 4b:** Effects of the job crafting intervention program on work-related outcomes for higher job crafting (job crafting scale > 5.00; *n* = 127).

	**Int (*****n*** = **67)**		**Cont (*****n*** = **60)**			**95% CI of estimates of fixed effects**			
	**EM**	**SE**	**EM**	**SE**	**Estimates of fixed effects**	**Lower**	**Higher**	**t**	***p***
**Work engagement**									
3-month	3.45	0.13	3.81	0.14	−0.04	−0.33	0.25	−0.27	0.79
6-month	3.31	0.13	3.60	0.14	0.03	−0.26	0.31	0.19	0.85
Pooled					0.01	−0.18	0.20	0.09	0.93
**Job crafting**									
3-month	5.62	0.09	5.63	0.09	0.15	−0.11	0.41	1.17	0.25
6-month	5.44	0.09	5.58	0.09	0.02	−0.28	0.33	0.15	0.88
Pooled					0.02	−0.14	0.18	0.24	0.81
**Task crafting**									
3-month	5.80	0.10	5.73	0.10	0.18	−0.09	0.44	1.32	0.19
6-month	5.55	0.10	5.68	0.10	−0.01	−0.31	0.28	−0.09	0.93
Pooled					0.005	−0.16	0.17	0.05	0.96
**Relational crafting**									
3-month	5.54	0.11	5.60	0.11	0.10	−0.23	0.42	0.58	0.56
6-month	5.41	0.11	5.58	0.11	−0.02	−0.39	0.36	−0.08	0.94
Pooled					−0.002	−0.20	0.19	−0.02	0.98
**Cognitive crafting**									
3-month	5.50	0.13	5.55	0.13	0.19	−0.20	0.58	0.98	0.33
6-month	5.34	0.13	5.48	0.14	0.09	−0.34	0.52	0.43	0.67
Pooled					0.05	−0.18	0.29	0.45	0.65
						**95% CI of Cohen's** ***d***			
	***n***	**Cohen's** ***d***		**Lower**			**Higher**		
**Work engagement**									
3-month^*^	114		0.01		−0.36		0.37		
6-month^**^	102		0.04		−0.35		0.43		
**Job crafting**									
3-month^*^	113		0.24		−0.13		0.61		
6-month^**^	102		0.01		−0.38		0.40		
**Task crafting**									
3-month^*^	114		0.22		−0.15		0.58		
6-month^**^	102		−0.04		−0.43		0.35		
**Relational crafting**									
3-month^*^	113		0.14		−0.23		0.51		
6-month^**^	102		−0.03		−0.42		0.36		
**Cognitive crafting**									
3-month^*^	114		0.22		−0.14		0.59		
6-month^**^	102		0.08		−0.31		0.47		

Int, Intervention; Cont, Control; EM, Estimated means; SE, Standard errors.

* Cohen's d between baseline and 3-month follow-up survey were based on the score of the respondents who completed both surveys.

** Cohen's d between baseline and 6-month follow-up survey were based on the score of the respondents who completed both surveys.

In addition, there was an error in the Section **Results**, “*Effects of the Job Crafting Intervention Program on Each Outcome Variable*.” “0.16 (95% CI: −0.09 to 0.41) at 3-month follow-up and 0.04 (95% CI: −0.22 to 0.31) at 6-month follow-up” should have been “0.15 (95% CI: −0.10 to 0.40) at 3-month follow-up and 0.03 (95% CI: −0.24 to 0.29) at 6-month follow-up.” The corrected paragraph is included below:

“Table 2 presents the means and SDs of the outcome variables at baseline, 3-month, and 6-month follow-up in the intervention and the control groups. Table 3 shows the estimated effects of the job crafting intervention program on the outcome variables based on the mixed model analyses as well as effect sizes (Cohen's *d*). None of the growth models including random effects converged; thus, only fixed effect results from the model including are reported here. Regarding the variance model, the model that included random intercept was adopted. The job crafting intervention program showed a non-significant effect on work engagement. The effect sizes for work engagement were small, with values of 0.15 (95% CI: −0.10 to 0.40) at 3-month follow-up and 0.03 (95% CI: −0.24 to 0.29) at 6-month follow-up. The job crafting intervention program had a non-significant effect on job crafting, which effect size was also small.”

The authors apologize for these errors and state that this does not change the scientific conclusions of the article in any way. The original article has been updated.

